# Supplementary figures and images for: Inhibitory feedback from the motor circuit gates mechanosensory processing in Caenorhabditis elegans
Source: PLoS Biol. 2023 Sep 21;21(9):e3002280. doi: 10.1371/journal.pbio.3002280 (PMC10617738; doi:10.1371/journal.pbio.3002280)

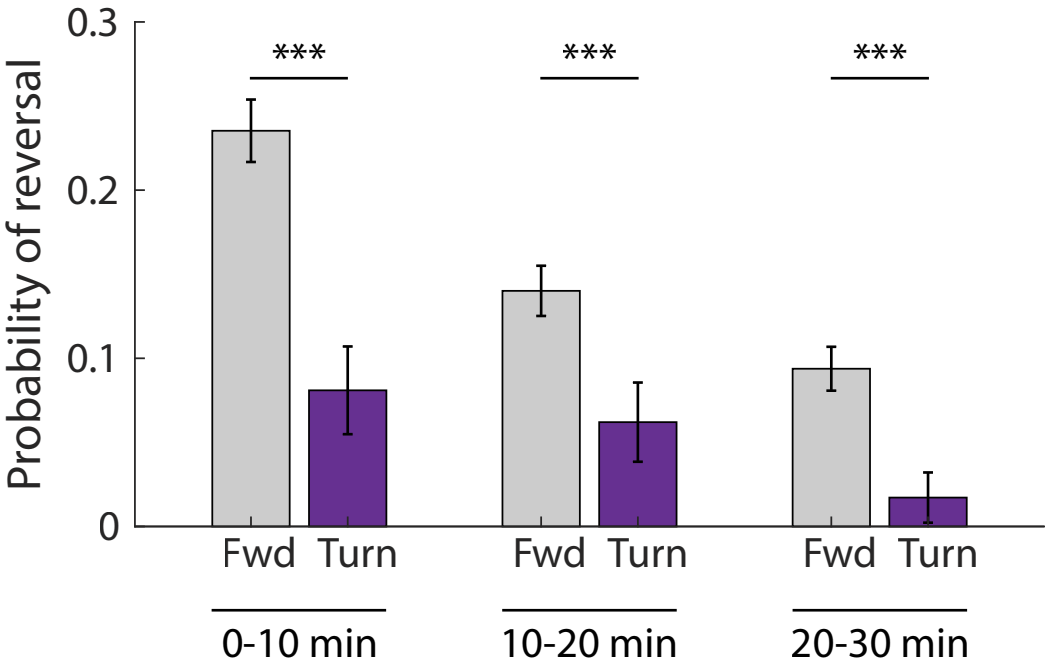

Supplement: S1 Fig — Probability of evoked reversal in response to optogenetic stimulation to gentle-touch mechanosensory neurons (Pmec-4::Chrimson) is calculated for three different portions of the 30-min experiment. Habituation is visible, but the relative difference in reversal probability persists. Error bars show 95% confidence intervals of the population proportions; *** indicates p<0.001 via two-proportion Z-test. Exact p values for all the statistical tests are listed in S1 Table. N = 2,006, 420, 2,077, 403, 1,919, and 291 stimulation events from left to right. The number of assay plates for forward and turn context are N = 29 and 47, respectively. This figure is a reanalysis of measurements presented in [19]. All data underlying this figure can be found at https://doi.org/10.25452/figshare.plus.23903202. (PDF) [file pbio.3002280.s001.pdf]

**A**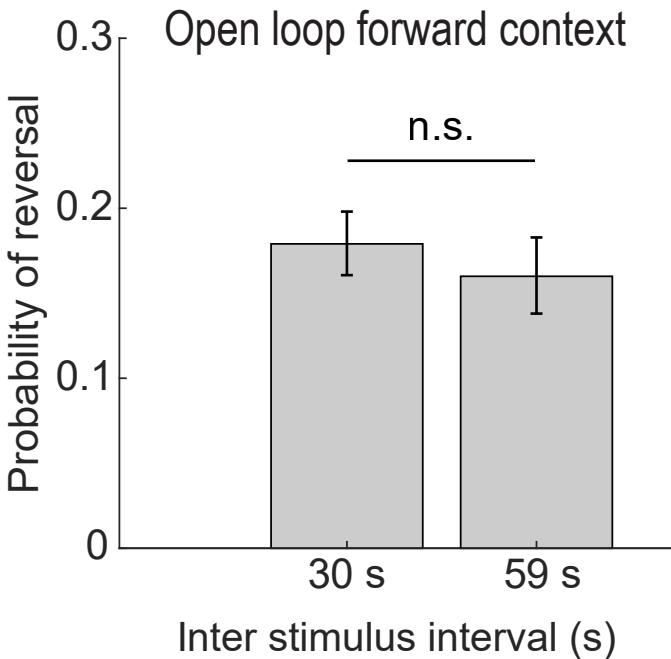**B**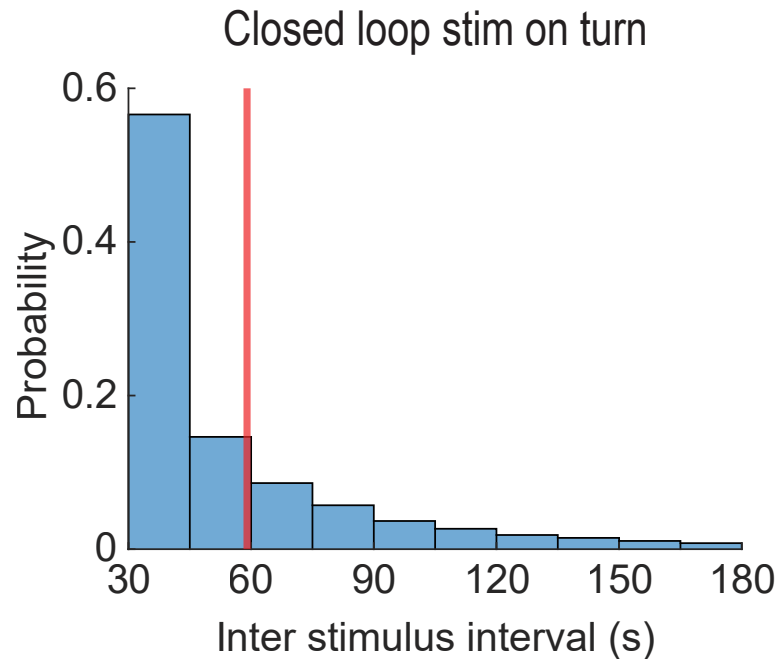

Supplement: S2 Fig — (A) Animals expressing Chrimson in their gentle-touch mechanosensory neurons were optogenetically stimulated in open loop every 30 s or 59 s. Only responses to stimuli delivered during forward locomotion are included. These are new experiments not previously reported. N = 1,631 and 1,094 stim events for 30 s and 59 s inter-stimulus interval assays. We used four plates for both 30 s and 59 s inter-stimulus interval assays. Error bars show 95% confidence intervals of the population proportions, and p value via two-proportion Z-test is 0.196. (B) 59 s (vertical red bar) is the mean inter stimulus interval (ISI) experienced by worms in the closed-loop turn-triggered stimulus experiments previously presented in [19]. The ISI is not constant because it depends on when the worm turns. The distribution of the ISI experienced by worms during those experiments in Fig 1 is shown in blue. All data underlying this figure can be found at https://doi.org/10.25452/figshare.plus.23903202. (PDF) [file pbio.3002280.s002.pdf]

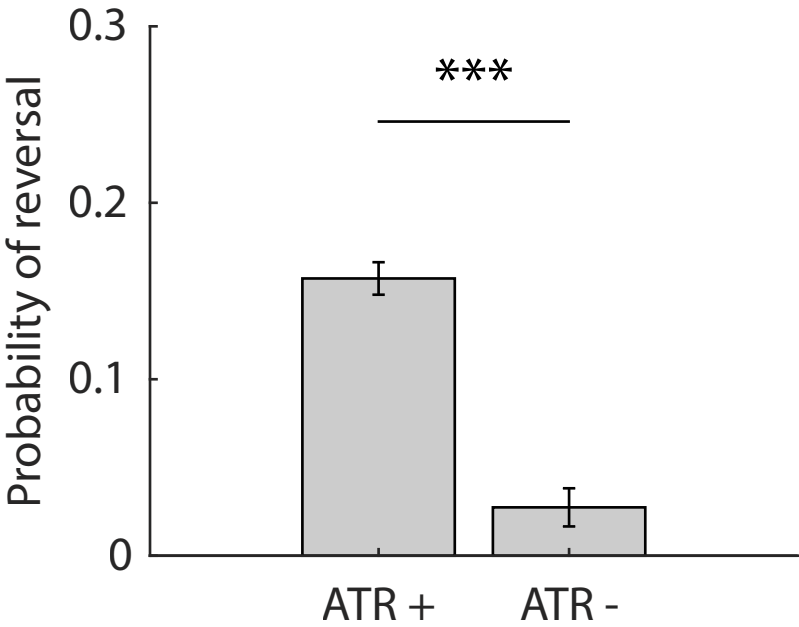

Supplement: S3 Fig — Animals that express chrimson in the touch receptor neurons were grown in the presence or absence of the necessary co-factor all-trans retinal (ATR) and exposed to 80 μW/mm2 intensity red light. The 95% confidence intervals for population proportions are reported. Two sample Z-test was used to calculate significance; *** indicates p<0.001. The exact p value is listed in S1 Table. The number of stimulus events for each condition (from left bar to right bar) are: 6,002 and 876. The number of assay plates for ATR + and ATR − conditions are 29, 4. Note that the ATR + condition was previously reported in [19] and also appears in Fig 1C and 1D. The ATR − condition was recorded contemporaneously, but is presented here for the first time. All data underlying this figure can be found at https://doi.org/10.25452/figshare.plus.23903202. (PDF) [file pbio.3002280.s003.pdf]

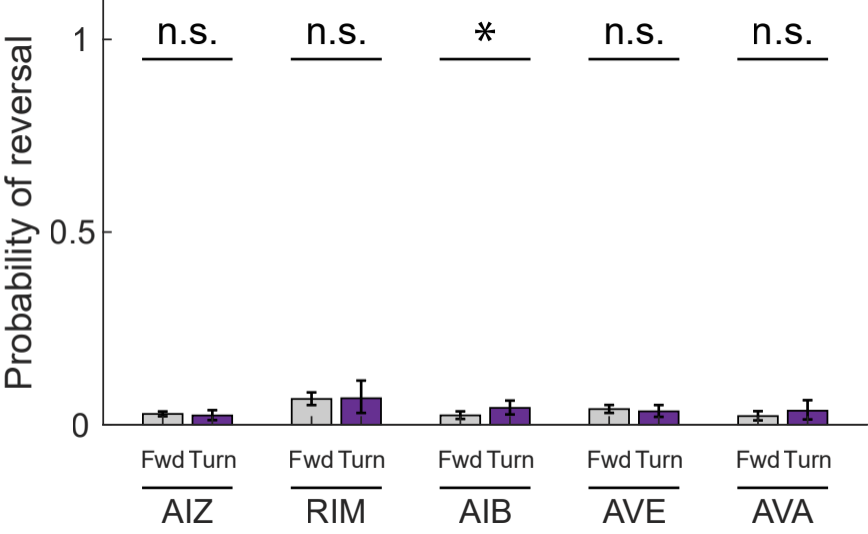

Supplement: S4 Fig — These are control experiments corresponding to the experiments presented in Fig 2B. Baseline reversal probabilities for each strain in each condition are measured by shining a low-intensity control stimulus. Three seconds of only 0.5 μW/mm2 of red light illumination (neuron AVE and AVA) or 2 μW/mm2 of blue light illumination (neuron AIZ, RIM, and AIB). The 95% confidence intervals for population proportions are reported. Two proportion Z-test was used to calculate significance; p value for AIZ, RIM, AIB, AVE, and AVA stimulation group is 0.596, 0.936, 0.045, 0.565, 0.262, respectively. The number of stimulus events for each condition (from left-most bar to right-most bar) are: 2,646, 583, 883, 131, 867, 527, 1,406, 490, 626, and 220. The number of assay plates for forward and turn context for neurons from left to right are 16, 27, 12, 19, 4, 24, 8, 16, 8, and 20. All data underlying this figure can be found at https://doi.org/10.25452/figshare.plus.23903202. (PDF) [file pbio.3002280.s004.pdf]

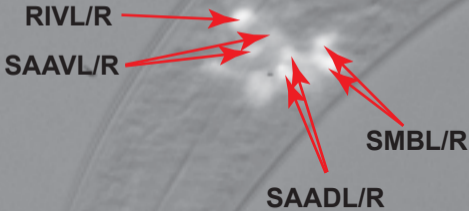

Supplement: S5 Fig — Fluorescence/Bright field, merged image of AML496 worms showing the expression of eGFP driven by lim-4 promoter using (Plim-4::gtACR2::SL2::eGFP) expression vector. eGFP can be seen in the neurons RIV, SMB, and SAA. (PDF) [file pbio.3002280.s005.pdf]

Chrimson in  
touch neurons

gtACR2 in  
RIV, SMB, SAA

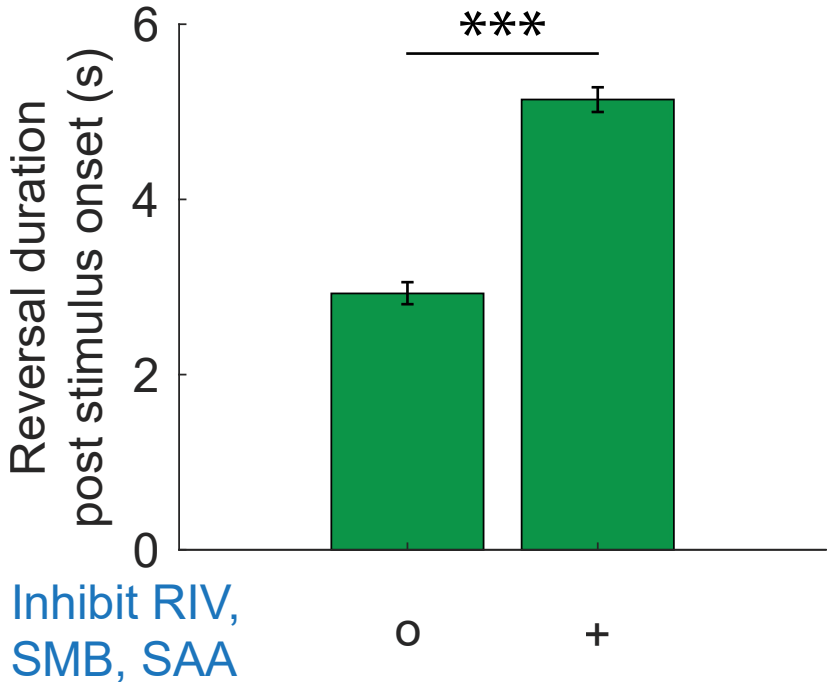

Supplement: S6 Fig — Same experiment as in Fig 3, but in a transgenic background that also expresses Chrimson in the mechanosensory neurons. Results are consistent with Fig 3. Worm spent more time reversing when the RIV, SMB, and SAA neurons were inhibited compared to when a control stimulus intensity was used. Error bars represent 95% confidence intervals; *** indicates p<0.001 via two-proportion Z-test. The exact p value is listed in S1 Table. The number of stimulus events for mock and experimental conditions are 1,168 and 1,364, respectively. The number of assays was N = 12. All data underlying this figure can be found at https://doi.org/10.25452/figshare.plus.23903202. (PDF) [file pbio.3002280.s006.pdf]

A

Chrimson in  
touch neuronsgtACR2 in  
RIV, SMB, SAA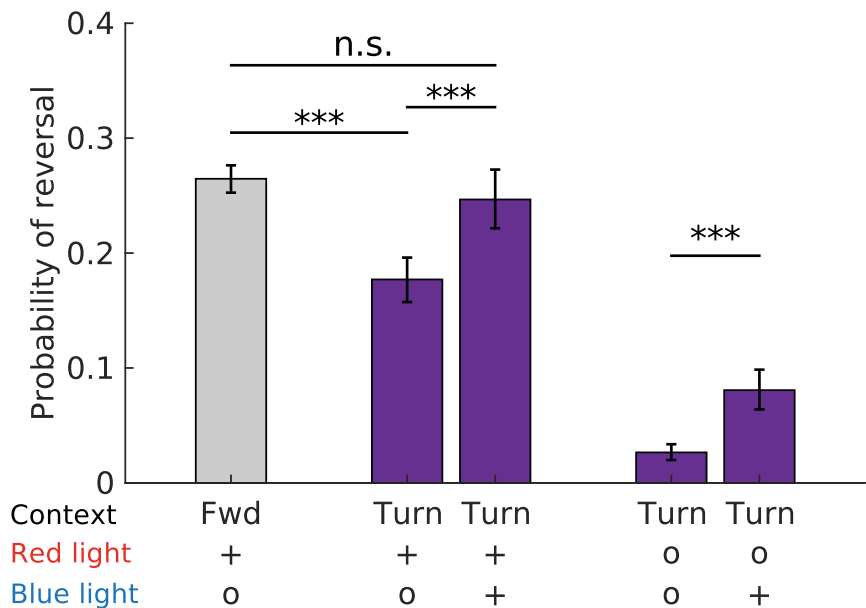

B

Chrimson in  
touch neurons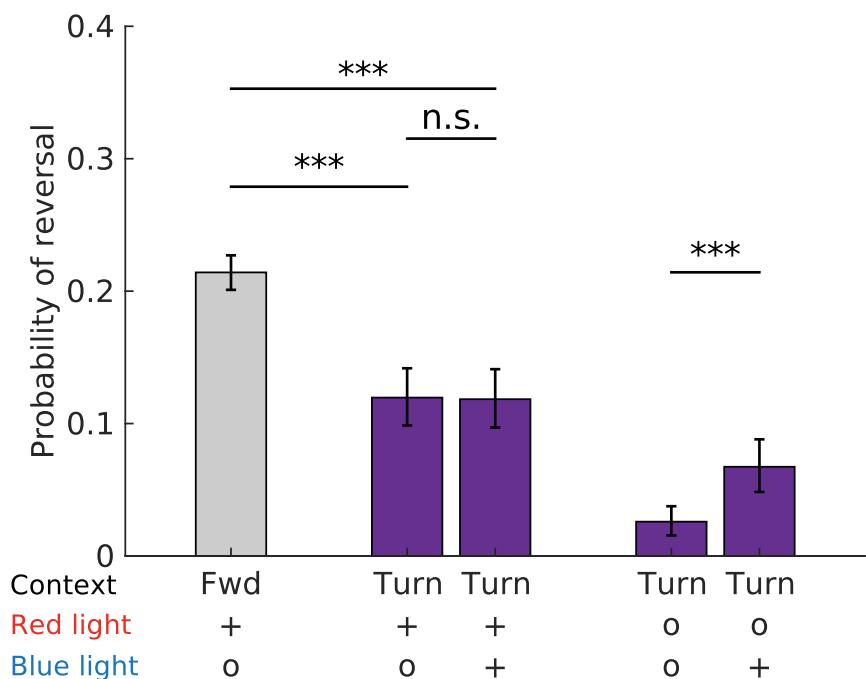

C

gtACR2 in  
RIV, SMB, SAA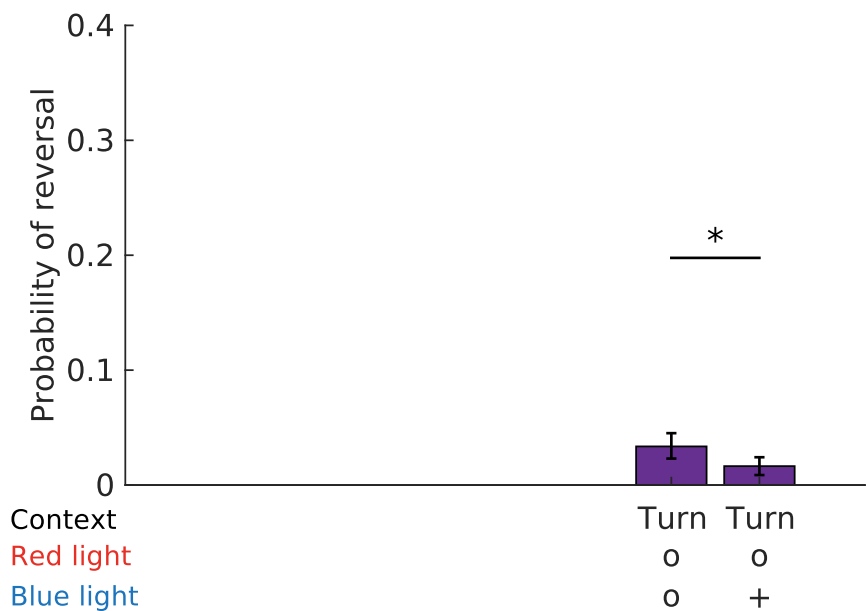

Supplement: S7 Fig — (A) Probability of reversals when either touch neurons are activated, or RIV, SMB, and SAA are inhibited, or both simultaneously; during either forward movement or turn onset. First three bars are same as in Fig 4. Touch neurons express Chrimson and are activated with red light. RIV, SMB, and SAA expressing gtACR2 are inhibited with blue light. Strains are listed in Table 1. The 95% confidence intervals for population proportions are reported. N = 5,381, 1,525, 1,115, 1,961, and 954 stim events, from left to right. The number of assays from left to right bars are: N = 8, 16, 16, 8, and 15. (B) Same experiments were repeated in a strain that expressed Chrimson in the gentle-touch mechanosensory neurons, but no inhibitory opsins. N = 3,722, 903, 794, 772, and 579 stim events. The number of assays from left to right bars are: N = 6, 12, 15, 15, and 16. (C) Same experiments are shown for animals that only express inhibitory opsin gtACR2 in RIV, SMB, and SAA, but no Chrimson. N = 1,041 and 1,033 stim events. The number of assay is: N = 16; *** indicates p<0.001, “n.s.” indicates p>0.05 via two-proportion Z-test. Exact p values for all the statistical tests are listed in S1 Table. All data underlying this figure can be found at https://doi.org/10.25452/figshare.plus.23903202. (PDF) [file pbio.3002280.s007.pdf]

A

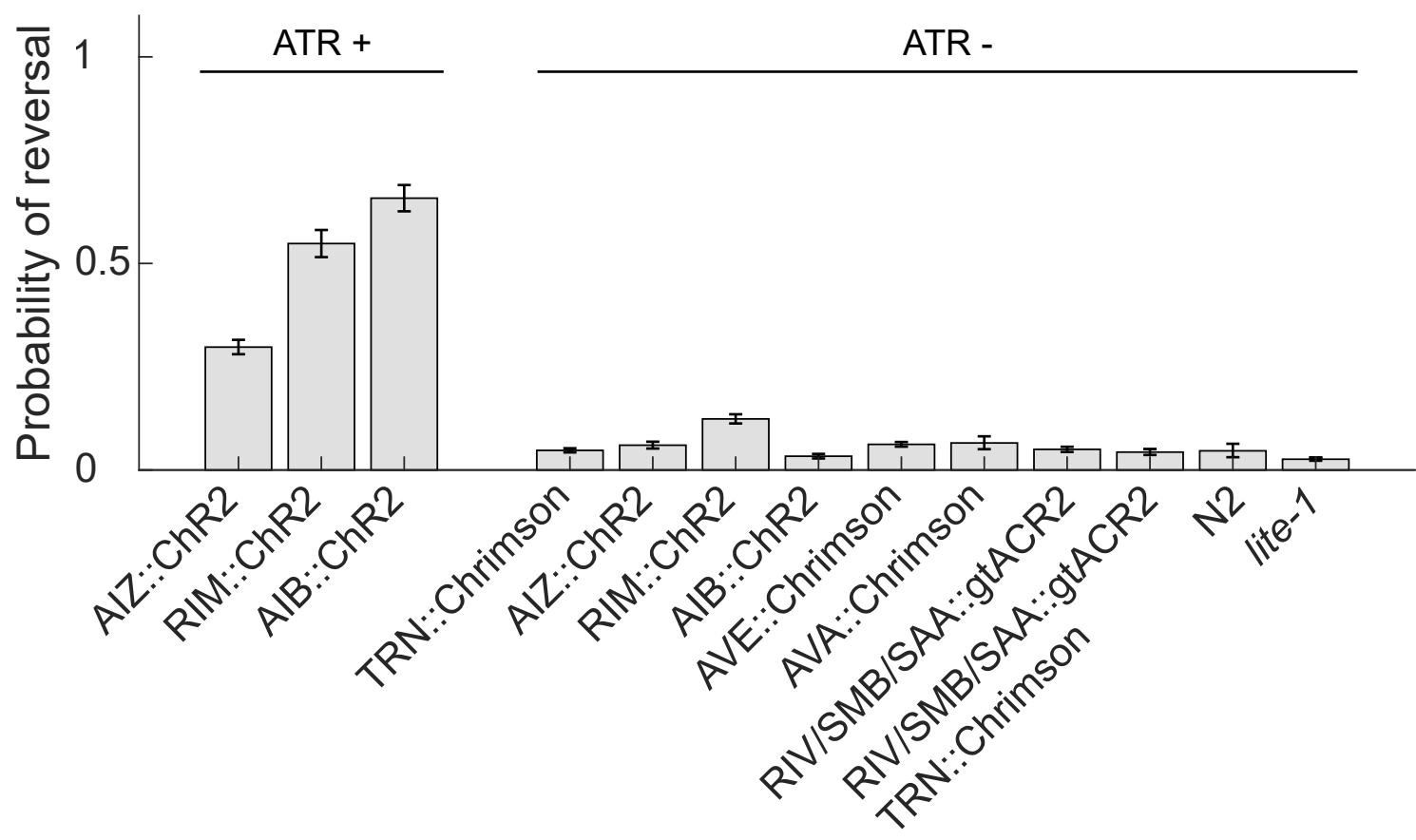

B

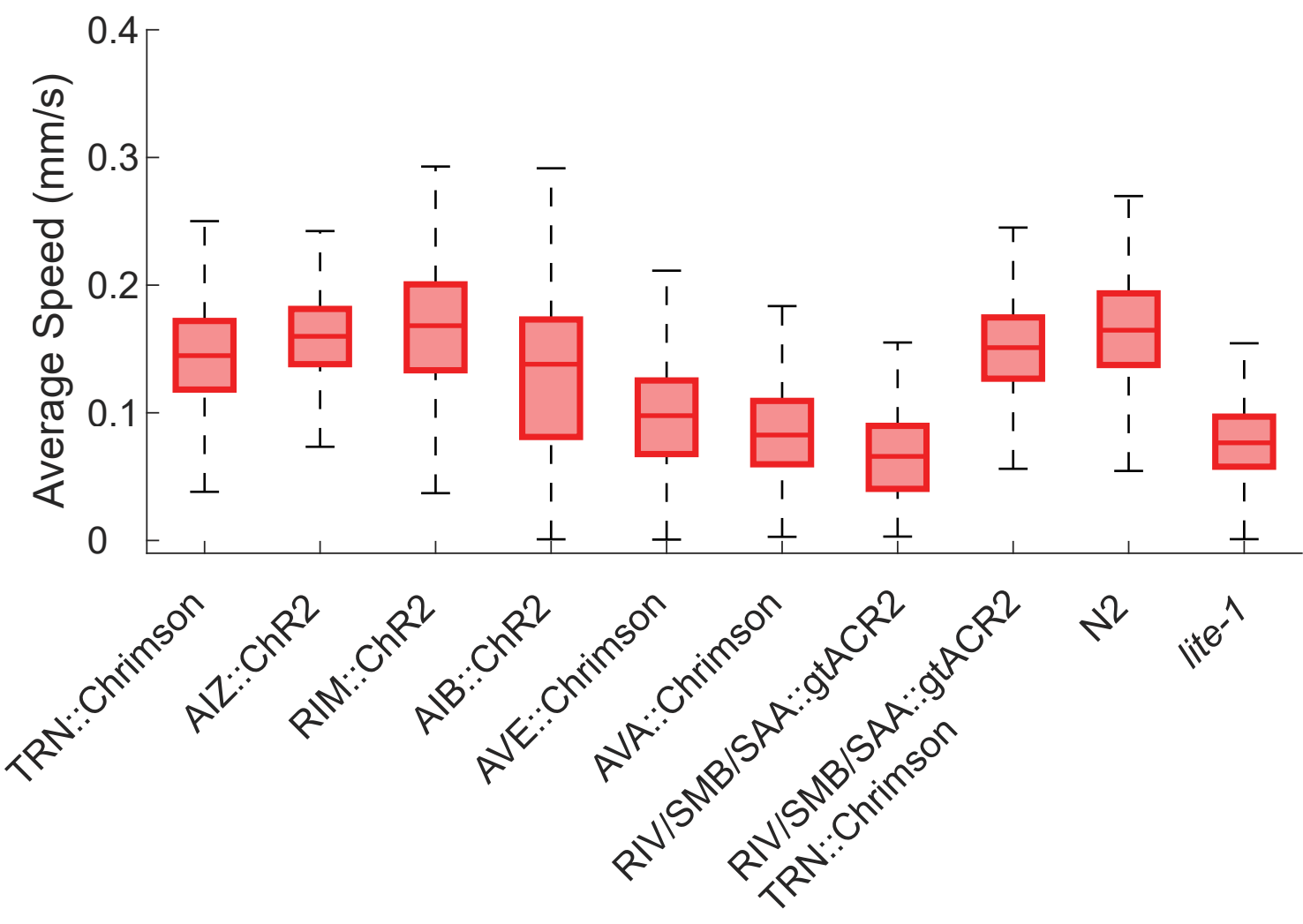

Supplement: S8 Fig — (A) To characterize endogenous sensitivity to blue light, blue light-evoked reversal probability is measured for different strains with and without the all-trans retinal (ATR) co-factor needed for optogenetic proteins. The 300 μW/mm2 blue light intensity used here, is less than that reported to evoke the animal’s endogenous blue light response [64]. Only those strains that express ChR2 are measured on retinal (ATR+, left, N = 2,612, 883, 880 from left to right, same as Fig 2B), while all strains, including the Chrimson strains, are measured in the off-retinal condition (ATR-, right, N = 6,564, 3,213, 3,365, 3,867, 7,006, 993, 4,516, 3,324, 646, and 6,470 from left to right). Error bars show 95% confidence intervals for population proportions. We include a lite-1 mutant and wild-type N2 for comparison because our transgenic strains include a mix of both wild-type and lite-1 backgrounds. (B) Average speed of each strains used in this work are shown N = 1,654, 564, 1,065, 654, 983, 1,099, 1,251, 837, 1,706, and 1,952 from left to right. All data underlying this figure can be found at https://doi.org/10.25452/figshare.plus.23903202. (PDF) [file pbio.3002280.s008.pdf]
