# Supplementary material for: Inhibitory feedback from the motor circuit gates mechanosensory processing in Caenorhabditis elegans
Source: PLoS Biol. 2023 Sep 21;21(9):e3002280. doi: 10.1371/journal.pbio.3002280 (PMC10617738; doi:10.1371/journal.pbio.3002280)
Supplement: S1 Text — (PDF) [file pbio.3002280.s016.pdf]

S1 Text. Additional control experiments show that blue-light alone cannot restore mechanosensory evoked reversals.

We sought to rule out alternative explanations for why shining blue light during turns may cause an increase in reversals. It is known that the nominally red-light sensitive Chrimson can also be mildly activated by blue light [1]. We therefore tested whether the increase in responsiveness to the mechanosensory stimulus was the result of blue-light activation of Chrimson expressed in the touch neurons.

Consistent with mild blue-light activation of Chrimson, shining only blue light on animals expressing Chrimson in the touch neurons (S7A Fig and S7B Fig far right bar) but not on animals expressing only gtACR2 in the turning neurons (S7C Fig far right bar) caused a significant increase in the probability of reversals compared to no stimulation (second to right bar). However, this mild blue-light activation of Chrimson is insufficient to explain the increase in reversal probability we observed when inhibiting turning neurons via gtACR2. Even when shining both blue and red light on animals that lack the inhibitory opsin gtACR2, but do contain Chrimson in their touch neurons, we still observed a large and significant reduction in the likelihood of reversing in response to stimuli delivered during turns compared to delivered during forward locomotion (S7B Fig middle bar, compared to far left bar). This suggests that it is the inhibition of neurons RIV, SMB and SAA that abolishes the turning-dependence of mechanosensory processing and not mild blue-light activation of the touch neurons. Further consistent with this view, adding blue light to red light in those animals that lack the inhibitory opsin does not significantly increase the probability of reversing (S7B Fig second compared to third bar).

A simple and fully consistent explanation is that our red light illumination strongly activates the touch neurons and that any additional blue light contributes only very modest additional activation to the touch neurons, and not enough to explain the increase we see when inhibiting RIV/SMB/SAA. Moreover, this modest additional blue-light activation of the touch neurons is only significant in control experiments without any red light. Taken together we conclude that inhibition of the turning neurons, and not mild blue-light activation of Chrimson, is responsible for abolishing the turning dependence of the mechanosensory response.

Supplementary Text References:

1. Klapoetke NC, Murata Y, Kim SS, Pulver SR, Birdsey-Benson A, Cho YK, et al. Independent optical excitation of distinct neural populations. Nature methods. 2014;11(3):338–346. doi:10.1038/nmeth.2836.
