# Supplementary material for: Inhibitory feedback from the motor circuit gates mechanosensory processing in Caenorhabditis elegans
Source: PLoS Biol. 2023 Sep 21;21(9):e3002280. doi: 10.1371/journal.pbio.3002280 (PMC10617738; doi:10.1371/journal.pbio.3002280)
Supplement: Attachment — Submitted filename: 20230721_Kumar_R2r_PLOSBio (1).pdf [file pbio.3002280.s017.pdf]

Kumar et al., 2023 Response to Reviewer Feedback

RE reviewer 2's comment starting "The figures are a bit weak"... the reviewer raises a few concerns about the presentation of the data, and I note the Response to Reviewers doesnt specify whether any changes have been made to address these (the R2R discussess the second half of this comment about animal-to-animal variation). For example R2 says "The bar charts are not very informative with respect to sample distribution, and without raster plot I do not get a good idea about the habituation to the stimuli"; "The legend says N number of stimulations from x plates, but not how many animals and stimuli per animals have been scored. What is the central tendency? 95% CI on the mean, median? Bootstrapping suggest it is the median, but I needed to guess. Exact p-values are not given, neither the degrees of freedom for their calculation (based on animals, plates, stimulations?)."

Can you provide a more thorough description of if/how you addressed these comments in the last revision, and point to where in the text/figures these changes have been made? This will facilitate the assessment of the revision. (I know that the underlying data provide on figshare may address aspects of this request, but we are ideally looking for changes in the manuscript)

We thank the editor for handling our manuscript.

Editor:

1) TITLE: We encourage you to spell out the genus name in the title. ""Inhibitory feedback from the motor circuit gates mechanosensory processing in Caenorhabditis elegans"

Fixed.

2) DATA: Thank you for providing the raw data related to your manuscript on figshare. Can you please reference this dataset in each relevant figure legend? For example, you can add the sentence "the data underlying this figure can be found at https://figshare.com/articles/dataset/kumar_etal_2022_dataset_tar/21699668"

Added.

We thank all four reviewers for thoughtful and timely comments and feedback.

Reviewer #1

Reviewer #1: In this study, the authors dissect the neural circuitry by which one C. elegans motor program (turning) influences sensorimotor transformations in another motor circuit (touch avoidance circuit). C. elegans has become a premier model for examining such problems, due to its well defined circuitry and the precision of the genetic/cellular perturbations that can be conducted. Building off of an elegant previous study, the authors here examine how defined turning neurons influence touch-elicited reversals. They first show that touch-induced reversals (elicited by opto stim of the touch sensory neurons) is inhibited both during reversal-associated turns and isolated turns (i.e. turns occurring outside of the context of the reversal behavioral sequence). Next, they perform the same experiment, but elicit the reversal at different nodes in the touch avoidance circuit. They find that the effect is maintained in some interneurons the lie downstream of the touch neurons,
but not everywhere. For example, stimulation of AVA command neurons elicits reversals regarding of turning status. This maps plausible locations where the turning circuit may act on the reversal circuit. They then optogenetically inhibit the turning neurons RIV/SAA/SMB and first show that this causes animals to have longer reversals, as expected. Next they should that this inhibition is sufficient to reverse the effects of turning on touch-elicited reversals. They propose an intriguing model where these motor programs inhibit one another, such that sensorimotor transformations are gated by ongoing motor state.

This is an exciting study that makes elegant use of excellent technologies uniquely available in the authors' lab. The study builds off of previous ones in the field, but adds significant value, arriving at a clear model. My suggestions (mostly non-essential) are below:

1. I think it would be nice to cite the corollary discharge papers related to RIM (Ji, Riedl, etc) in the Introduction, as well as the Discussion (where they are currently cited). This will provide readers with proper background info in case they are not familiar with the field.

Thank you for the suggestion. That citation was also already in the introduction, but we have added it to a second place in the introduction, so that it now appears three times in the manuscript.

2. I think that a direct -/+ ATR comparison for the TRN::Chrimson really belongs in Fig. 1, rather than being buried in supplement (would be nice to show -/+ side by side also). This should also be shown for the strain/effect in Fig. 3 since that involves exaggerated reversals (which in principle could be blue light-stimulated).

We have now added new experiments to new Supplementary Fig. S3, pasted below, showing the requested comparison, a direct -/+ ATR comparison for TRN:Chrimson response to *red* illumination.

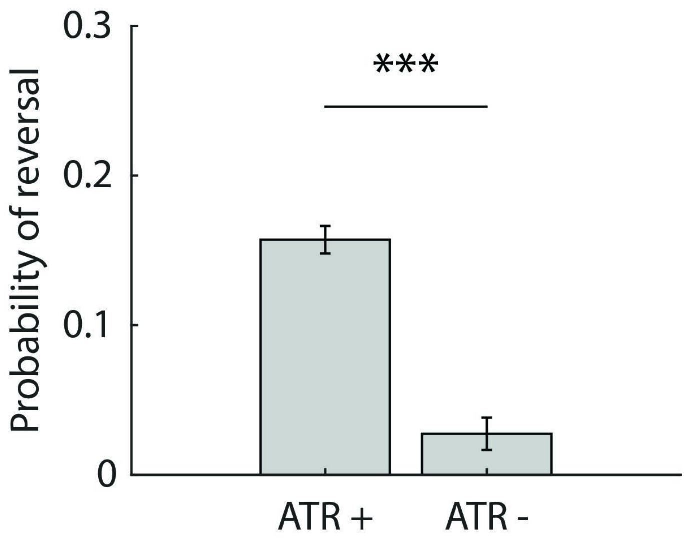

Regarding Fig 3: we already included the relevant ATR- blue-light illumination control in Supplementary Figure S8, under the condition labeled "RIV/SMB/SAA:gtACR2" As evident from that figure, we do not see any increase effect of blue light response in the ATR- animals compared to N2.

3. The specificity of the lim-4 promoter used for SAA/SMB/RIV expression is not discussed. I think it would be important to show an image of the neurons that it labels and list out the neurons in the main text. In case there are any additional neurons, it would be useful for this to be recorded in the paper.

We now include new Supplementary Fig S5, pasted below, showing the expression pattern for the Lim-4::ACR2 worms.

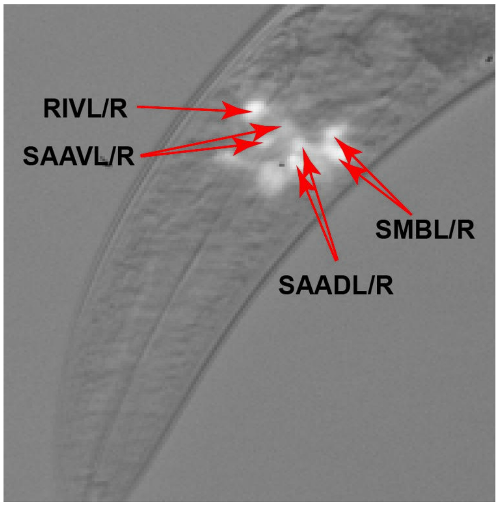

This is consistent with the fluorescent images of another strain with the same promoter shown in Fig 6a in (Wang et al., eLife 2020). We have also added the corresponding text:

"To express gtACR2 in RIV, SMB, and SAA, we used the lim-4, *promoter, as in (Wang et al., 2020) and confirmed the expression pattern using fluorescence microscopy, Supplementary Figure S5."*

4. The induction of reversals by TRN::Chrimson is fairly weak compared to the interneuron stimulations. Can the authors comment on this? Have they attempted experiments at higher light intensities and are the effects of turning on stimulated reversals the same? (non-essential)

Others have also observed that stimulation of interneuron AVA in particular is very efficient at evoking reversals. For example, Fig 1b from (Gordus et al., *Cell*, 2015) shows that optogenetic activation of AVA is roughly 5-fold more efficient at evoking reversal responses than activation of sensory neuron AWC. It is possible that interneurons are generally more efficient at driving motor outputs.

80 uW/mm^2 of red light is the highest illumination intensity that the current iteration of our instrument permits.

We do have some measurements at a lower intensity 40 uW/mm^2 from (Liu et al., PLoS Bio, 2022). A reanalysis of those experiments, pasted below, shows that even at 40 uW/mm^2, turning has a similar effect on stimulated reversals.

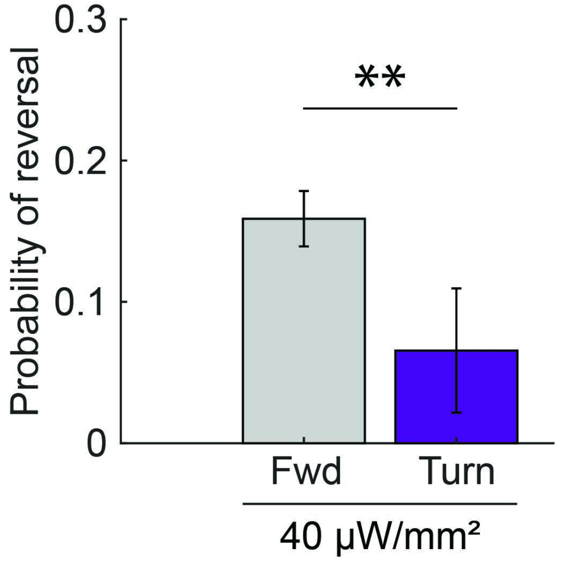

5. Does inhibition of RIV/SMB/SAA inhibit the induction of spontaneous reversals (in addition to extending reversal duration, as is shown in Fig. 3)?

We agree that this is an interesting idea. Probably the best way to explore this would be to use HisCl or some other method that is well-suited for chronic inhibition. In our

hands, and anecdotally in others, it has been challenging to get reliable and consistent long-term (~mins) inhibition using optogenetics. We will keep this in mind for future experiments.

6. In the dual-opto experiments, does the red light inhibit turning in a strain that only has lim-4::GtACR2? In other words, does the red light cross react with the blue opsin? Providing this type of information is useful for the field since other groups will likely become interested in dual-color optogenetics.

We have no reason to believe that the red light activates gtACR2. We did not notice any lim4::gtACR2 response under red light illumination in a fluorescent dissection microscope. gtACR2 should have virtually no excitation at the 620 nm red light wavelength that we use. See for example the action spectra for gtACR2 from (Govorunova et al., *Science*, 2015) Fig 1F, blue trace, pasted from that work below. We have added a blue arrow to indicate our illumination wavelength.

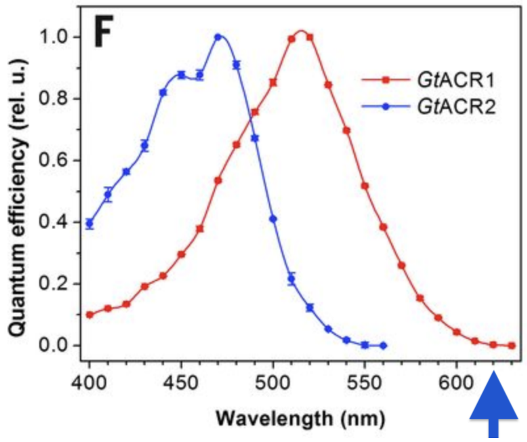

Photophysics is also in our favor here, as it would be very unusual for low energy far red photons to activate much higher energy transitions.

Reviewer #2

Reviewer #2: I am reviewing the paper ``Inhibitory feedback from the motor circuit gates mechanosensory processing in C.
elegans'' by Kumar et al.
In this manuscript the authors investigate how turning during C. elegans locomotion influences the touch response on the interneuron level. They observed that optogenetic stimulation of TRN-specific channelrhodopsins did not elicit a robust escape response when the animal was in the process of performing turn behavior. The authors identified that the turning-associated motor neurons (SMB, RIV, SAA) suppress processing of the touch response on the interneuron level. These observation and deduced wiring

diagram add significantly to the understanding of the systems-level regulation of a long-known sensory process. The research provides insight into more general mechanism of how motor-related signals can tune the neural dynamics, in the presence of mixture of contradictory sensory and motor signals. Thanks to automatizing the experimental pipeline, authors supported their hypotheses with the great amount of experimental data. The paper will greatly contribute to the field. I have several questions/comments.

We thank the reviewer for their enthusiasm.

I would suggest some improvements that should facilitate the understanding and its relation to the differentiate between sensory processing and sensitivity.
If I am not mistaken, the process under study is related to corollary discharge, in which information from the motorcircuit or downstream interneurons adjust sensory processing, eg. prepare the nervous system for an incoming change that needs to be suppressed. I miss a deeper introduction to these concepts and earlier work/reviews on this topic and the authors should include timely citations (PMID: 35690069, 33880993). The authors cite some of these, including Crapse et al, but the discussion of the TRNs in this review is quite confusing and probably different from what the authors study here, thus they should explain what specifically they cite this review for. For a more general introduction to the body brain feedback I suggest PMID 35687973. Is there evidence in general from the authors or in the literature that turns or body bends in general affect touch response?
To our knowledge we are not aware of any additional evidence in the literature that turns or body bends influence touch response. We also informally spoke with others in the field, including Marty Chalfie, and Miriam Goodman and they also were not aware of any findings in the literature prior to our first published observations in (Liu et al., 2018).

The observation that turns modulate TRN processing asks the question if turns towards the ventral side and turns towards the dorsal side are similarly affected. AVM and PVM are located on the ventral side and are thus asymmetrically affected by body bends. Does exaggerated bending during turns directly modulate the sensitivity/excitability of the TRNs? To differentiate between passive body bending that affects the sensitivity vs inhibitory feedback from the interneurons, the authors could perform experiments in animals with exaggerated body curvatures.

The evidence we present from RIV/SAA/SMB inhibition experiments shown in Figure 4 suggests that it is not the passive curvature that alters the TRNs sensitivity. In those experiments the worm has already begun turning and the animal has already adopted a high curvature shape, but the same activation of TRNs is able to evoke a reversal response.

From reading the manuscript I got the impression that the optogenetic stimulus was specifically patterned to activate anterior TRNs. but now I am not sure anymore. Posterior neurons are not discussed, but if the whole animal is illuminated how does optogenetic stimulation of the posterior touch neurons affect their results? Would PLM processing also be affected by turns, or would they still trigger escape and accelerate animal locomotion?

Both anterior and posterior TRNs are illuminated. We have now modified the text to make this more clear,

> *"In this work, we deliver whole body optogenetic illumination specifically when the animal is either moving forward, or turning, or reversing… and addresses each animal individually to shine light on their whole body…."*

And:

> *"Previously we reported that optogenetic activation of all six gentle-touch mechanosensory neurons delivered during forward locomotion…"*

We first discovered the turning dependence of touch response in the context of a tap assay (Liu et al., *eLife,* 2018), therefore it is appropriate to study this phenomena by activating all of the touch neurons simultaneously.

We agree with the reviewer that it would be interesting to explore anterior vs posterior contributions. In prior work we explored anterior vs posterior TRN activation for stimuli delivered during forward locomotion (Liu et al., PLoS Bio 2022). But performing anterior or posterior targeted stimulation during turns requires higher resolution spatial targeting because the animal is often in an omega shape and the head is much closer to the tail. We are interested in further improving our instrument to provide this capability in the future.

The turning associated motorneurons SMB, RIV and SAA were inhibited to activate the reversal response during turns. The rational was that they show increased Ca activity during turns in a freely behaving animal. Why have all three neurons been targeted and what is the response for each of them? Are they redundantly acting or is there a dominant player?

These neurons were targeted because of experiments first reported by (Wang et al., *eLife*, 2020) suggesting that they have a role in turning. Exploring them individually would be interesting but is currently beyond our capabilities. We do not know of single cell promoters for SMB, RIV or SAA and we are limited by the spatial targeting of our current instrument.

The figures are a bit weak in general. The bar charts are not very informative with respect to sample distribution, and without raster plot I do not get a good idea about the habituation to the stimuli (In Figure S1, a reanalysis is provided though). The legend says N number of stimulations from x plates, but not how many animals and stimuli per animals have been scored. What is the central tendency? 95% CI on the mean, median? Bootstrapping suggest it is the median, but I needed to guess. Exact p-values are not given, neither the degrees of freedom for their calculation (based on animals, plates, stimulations?). As the authors use a z-test as opposed to a t-test, they use the number of stimulations for their statistical comparisons.

We respond to each of these points in detail below. Here is an executive summary: We report the proportion of stimulus-events because this is the relevant metric, not an average within plates. Since we report a proportion across all stimulus-events it is not appropriate to report a mean or median. We calculate p-values via a z-test, as this is appropriate for comparing proportions. Bootstraps are not used, and this was a typo. Averaging within animals would be interesting but is impractical. And rasters would provide no additional information about how probability of reversals decreases due to habituation.

Here are the details:
The reviewer correctly notes that we treat stimulus-events as the fundamental unit, not animals or plates. Therefore the bars in, eg. Fig1c or 2b, provide the proportion of stimuli-events that resulted in a reversal, as listed on the y-axis. They do not report a mean or median. So the reader does not have to guess. Because we report the proportion, and not a mean or median, it is appropriate to report the analytically defined 95% confidence interval, and to calculate the significance of differences in our reported proportion using a z-test. In the paper we cite reference [63] https://www.itl.nist.gov/div898/handbook/prc/section3/prc33.htm which describes the z-test. We now clarify this new text in the methods.

> "*In our analysis, stimulus-events are the fundamental unit. Throughout the manuscript we report the proportion of all stimulus-events that result in a reversal, the number of stimulus events and the corresponding 95% confidence interval, calculated analytically. To reject the null hypothesis that two empirically observed proportions are the same, we use a two-proportion Z-test and report a p-value [63].*"

The reviewer expects that we should first be averaging responses within a plate, and then reporting the average of averages across plates. We assume this is why the reviewer expects a mean or median, or some central tendency. In our case it is not appropriate to average within plate. Recall our instrument provides closed-loop stimulation to individual animals based on their own individual behavior. Consequently,

even different worms on the same plate experience different stimuli. Because the worm's experience is not determined by the plate, we argue it is not appropriate to average within plate.

In the captions, we do list the number of plates for each assay. If this is confusing, we are happy to remove plate information entirely, because we don't think it's that relevant. We only included the number of plates because it provides some information about the approximate number of worms measured. As we describe in the methods,

> *"All experiments are performed on plates containing approximately 30 to 40 animals."*

So if there were 10 plates, the reader could then know that this corresponds to order a few hundred animals.

We agree with the reviewer that it would be interesting and relevant to report the average response probability by animal because it would provide insights into animal-to-animal variability. But this is currently impractical at the desired throughput because it would require us either to measure animals in isolation one-at-a-time (at dramatically lower throughput) or to confidently track animals through collisions or as they wander in and out of our field of view, which is beyond our current technical capabilities. Indeed, to our knowledge this is beyond the capabilities of any reported method in the field.

Regarding habituation, we provide Figure S1, pasted below, which we argue, and the reviewer acknowledges, shows evidence of habituation.

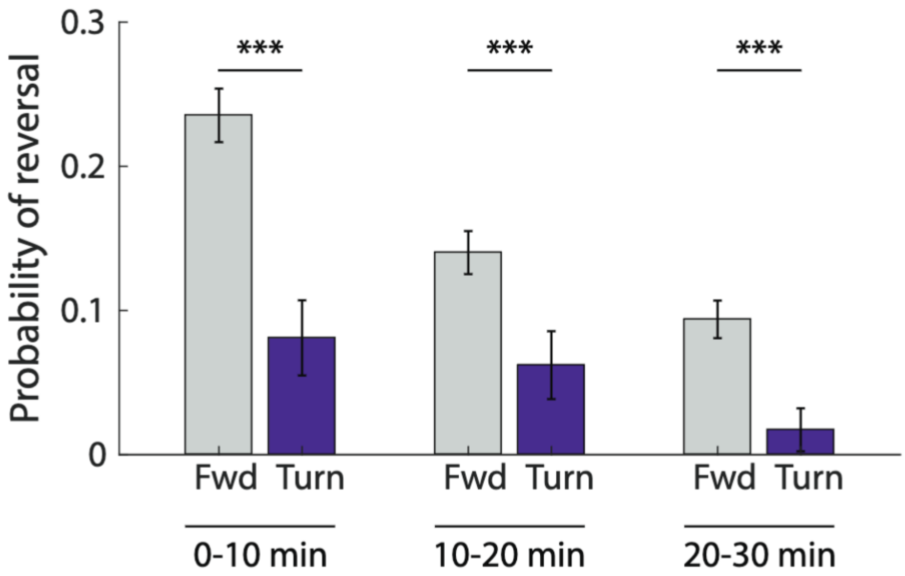

The reviewer suggests raster plots for inspecting how the proportion of stimulus-events that result in a reversal decreased over time due to habituation. We argue that showing

a raster plot for these time bins conveys no additional information about the probability of reversing because reversals are a binary event in this analysis. An analogy to coin flips may be informative. If I flip a coin 100 times, it is sufficient to report that the proportion of heads was 0.5 for N=100. A raster of those 100 flips does not provide any additional information. (Of course a raster might be useful for studying timing of the coin flip/reversal, or how long the flip/reversal took, but that's not under discussion here.) For this reason we hesitate to include a raster.

We apologize for the errant reference to a bootstrap. That was a typo left over from an earlier version of the manuscript. We have now removed the offending text. No bootstraps are used anywhere in this work.

At the reviewer's request we are now including a Supplementary Table S3, that reports exact p-values for all figures. In the caption we continue to use the *** convention, for brevity:

> "*** *indicates p < 0.001 via two sample z-test* "

One would wonder how the stimulus-response actually varied between individual plates and animals.

We agree it would be interesting to explore animal-to-animal variation. This is currently impractical at the desired throughput because it would require us either to measure animals in isolation one-at-a-time (at dramatically lower throughput) or to confidently track animals through collisions or as they wander in and out of our field of view, which is beyond our current technical capabilities.

The authors should also expand their discussion on why this escape response needs to be suppressed.
The authors state that: ``Suppressing mechanosensory-evoked reversals during turns may be part of a predator avoidance strategy. Turns are an important part of the C. elegans escape response, and by preventing turns from being interrupted prematurely, the animal may be ensuring that the escape response continues to completion.''. but this argument seems not very intruiging as the animal is might encounter a new threat which justifies a full sensory response. What I am trying to say is the the successful completion of the escape response into a new threat does not help the animal to survive. Is it expected that TRNs activate during omega turns? Is there a precedence for this? If so, this would make a strong physiological link between the motor command circuit and the suppression of a sensory stimulus. I don't think there is evidence in the literature that TRNs activate during omega turn, but if the authors had this data, it would make a strong point.
These are interesting points. Unfortunately we don't have this data. And we hesitate to speculate further about potential arguments of evolutionary fitness.

In the last decade, there has been some work supporting the hierarchical organisation of neural network in C. elegans, (e.g. PMID: 31786012), where forward-backward locomotion switch is at the highest organisational level (with AIB neuron as a representative), and with head bends/turns at lower organisational level. Can authors comment, even hypothetically, how their model relates to that? Is it contradictory to hierarchical organisation model?

We hesitate to speculate on this subject because we are unsure how our experiments fit into a hierarchical framework.

The authors should discuss the limitations of their optogenetic approach, e.g. lack of physiological depolarization, no locally confined signals, e.g. the whole axon and dendrite are being stimulated. Compartmentalized activity has a strong role in sensory and interneuron processing, as discussed in PMID: 22722842, 34533987, 35687973, 30609235. This is important especially during bends where local strains can locally activate and suppress neuronal activity through stretch-activated and compression activate channels (PMID 34533987), without structurally determined compartment boundaries.

In (Liu et al., *eLife*, 2018) Fig 2, pasted below, we show extensively that optogenetic whole animal stimulation recapitulates behavior responses seen in tap.This is strong evidence that the optogenetic stimulation is reasonably physiological.

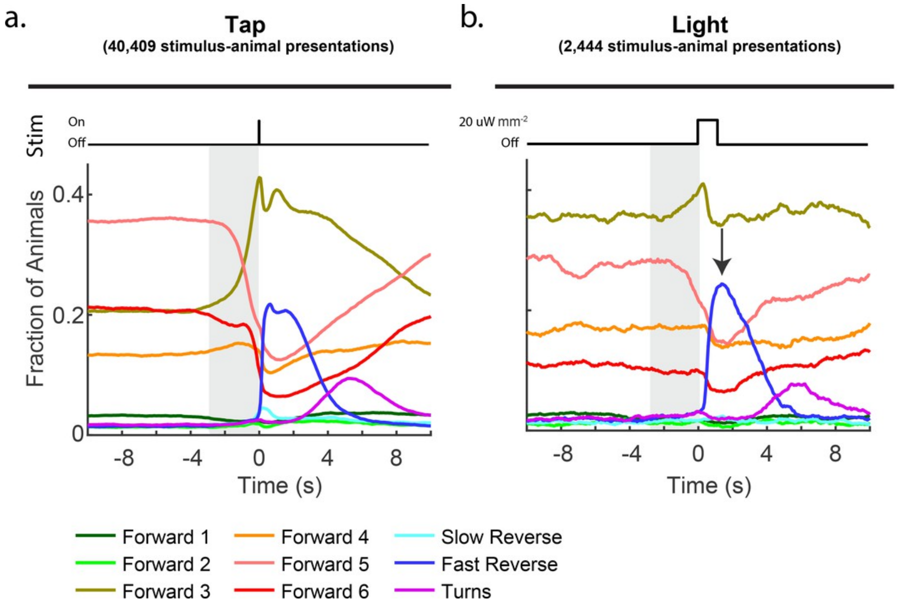

The reviewer suggests limitations, but all those listed apply to all optogenetic investigations in the field. I am not aware of any work in *C. elegans* that targets optogenetic stimulation to compartments within a neuron, with one exception: a single low-throughput experiment in one of our previous works (Leifer et al., *Nature Methods*,

2011, Fig 1b). It would not be practical to adapt that low throughput sub-compartment approaches for this high throughput application.

Specific points:
1.end of the first page: ''modulate mechanosensory response.'' This is very general but the references given only refer to TRNs. I suggest to either change to touch response or include reference that underline the general statement, such as proprioception, nociception etc....
Touch response is a mechanosensory response, so we suggest that the statement is accurate and justified.

2. it is not really clear what the authors mean by this sentence: ''this may explain why behavior-related neural activity patterns are seen across the brain in mice, fly and worms, including in nominally sensory areas''
We have updated the language and now clarify,

> *"Our findings add to a growing body of evidence suggesting that behavior information is necessary for sensory processing. The brain's presumed need to access both types of information in the same place may explain why behavior-related neural activity patterns are seen across so many brain areas in mice, fly and worms, including in nominally sensory areas…"*

3. The authors should discuss that rig-3 is not exclusively expressed in AVA, which could have consequences on their results. AVA-specific expression of ChR can be found in 10.1038/s41592-023-01836-9

We show in Table 1, partially pasted below, that *rig-3* also has non specific expression in other neurons I1, I4, M4, and NSM.

AML17	AVA	I1, I4, M4, and NSM [57]
[illegible]	[illegible]	

And we have now added the following text:

> *"Some strains have additional expression in other neurons, listed in Table 1. For example the promoter rig-3 is widely used to study AVA (Piggott et al., Cell, 2011; Shipley et al., Front Neur Circuits, 2014; and Gordus et al., Cell 2015), as it is here, despite also having off-target expression in pharyngeal neurons I1, I4, M4, and NSM neurons (Schmitt et al., PLOS One, 2012)."*

The conventional wisdom in the field is that because the other neurons expressed by *rig-3* are in the pharynx and their downstream connections are to pharyngeal neurons

and pharynx muscles, these neurons are allegedly unlikely to relate to the locomotor activity under investigation. Wiring diagram pasted from NemaNode is shown below: https://nemanode.org/?db=complete&ds=white_1986_whole&c=type&la=concentric&tc=1&te=1&sl=1&si=0&se=0&sp=1&sa=0&in=I1__I4__M4__NSM&h=G1&cor=I1_520_80__I5_604_296__RIP_385_296__I2_823_296__MI_713_296__M2_906_-40__M3_677_-51__MC_275_296__NSM_677_80__I3_494_296__I4_838_80__M1_1042_293__M4_993_80__I6_932_296__M5_1296_293__PM5_1212_293)

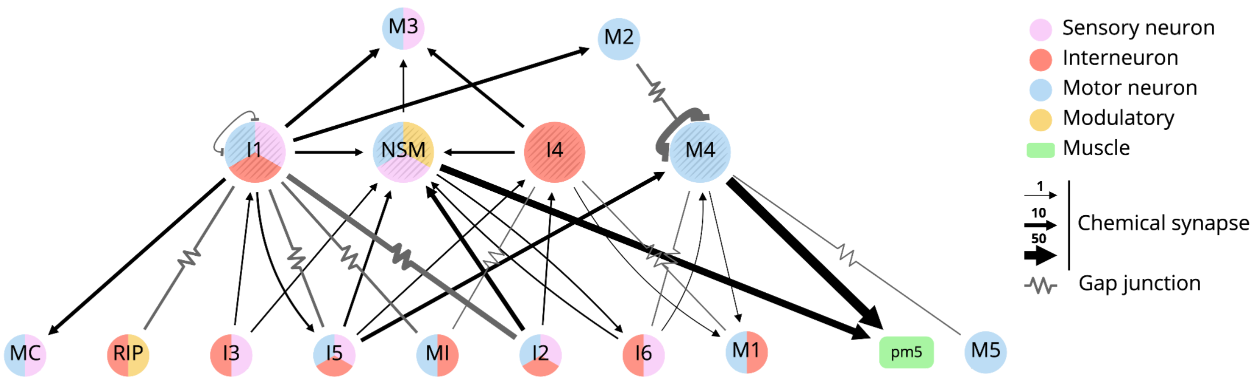

4. Authors need to explain what GtACR2 is?

We have now added clarifying text:

> *"We expressed the blue-light inhibitory opsin, gtACR2 (Govorunova et al., Science, 2015, Bergs et al., PLoS one, 2018), in these neurons and independently confirmed…"*

5. The notion that the authors claim that they not see a lite-1 dependent blue light activity is surprising and not expected from the literature. E.g. lite-1 has a strong effect in TRNs as well (e.g., doi: 10.1039/C6LC01165A). Are AML496 and 499 the only strains with wt lite-1 when blue light was used? the cleanest results would be to repeat these experiments in a lite-1 bg. Alternatively, the authors should discuss how the presence of lite-1 might influence their results.

We do not expect to see a blue light response because the blue light intensity we use, 300 uW/mm^2 is much lower than that reported to evoke a lite-1 response, as shown in Fig 1b from (Ward et al., Nat Neuro 2008), pasted below.We indicate the intensity we use with an added red arrow:

b

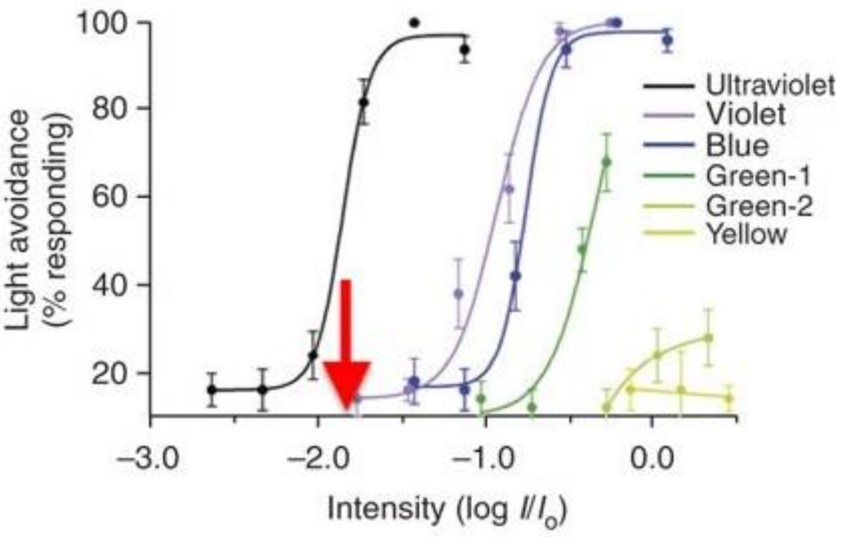

Using their conventions, I0 is defined to be 20,000 uW/mm^2, so for our intensity of ~300 uW/mm^2, log(I/I0) is ~-1.8.

We now add the following clarifying text to the caption in Supplementary Fig S8a:

> *"The 300 uW/mm^2 blue-light intensity used here, is less than that reported to evoke the animal's endogenous blue-light response (Ward et al., Nat Neuro 2008)"*

We emphasize that we systematically test for blue light response in every strain we used in the manuscript and present those measurements in Supplementary Fig S6a, pasted below.

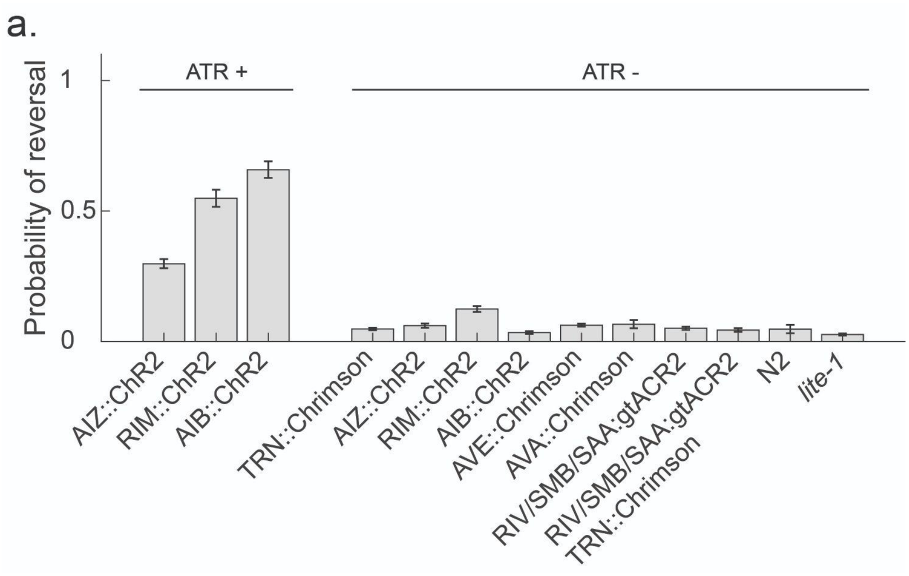

6. It is not clear which part of the worm has been illuminated during the turn? The whole worm or only the anterior part?

We now clarify:

> *"We then used our previously reported high-throughput closed-loop optogenetic delivery system (Liu et al., 2022) to stimulate the interneuron with 3 s whole-body illumination"*

And in other locations in the mansucript as well (now language is underlined):

> *"In this work, we deliver whole-body optogenetic illumination specifically when the animal is either moving forward, or turning, or reversing."*

And,

> *"In all cases we use a projector-based illumination system that tracks many individuals on a plate full of animals, segments them in real time, and addresses each animal individually to shine light on their whole-body,"*

7. It would significantly accelerate understanding if the authors would summarise in one sentence in the first paragraph in the results section what the experiment was, instead of only citing their previous paper.

Thank you for your suggestion. We have added the following sentence the paragraph immediately preceding the results section:

> *"Here we use this method to explore downstream mechanosensory processing by activating or inhibiting neurons associated with generating turns and reversals."*

Fig. 1: Why is the reversal probability to optogenetic stimulation so low? Wouldn't one expect a higher probability? Is the light intensity low or Chrimson not efficient to cause ChR2 levels of responses?

The light intensity for red light is 80 uW/mm^2. This is arguably modest, although it is clearly sufficient to activate the opsin, as shown in Fig 2b by the robust response to this light intensity for animals expressing Chrimson in AVA.

It is possible to generate pmec-4::Chrimson strains that are even more light sensitive, although the trends we see for the gating by turns is the same. Fig 4 of (Liu et al., PLOS Bio 2022), pasted below, compares light sensitivity of the strain we used, AML67 to a similar strain, AML470, made with a slightly different injection concentration and integration technique. The trends are the same.

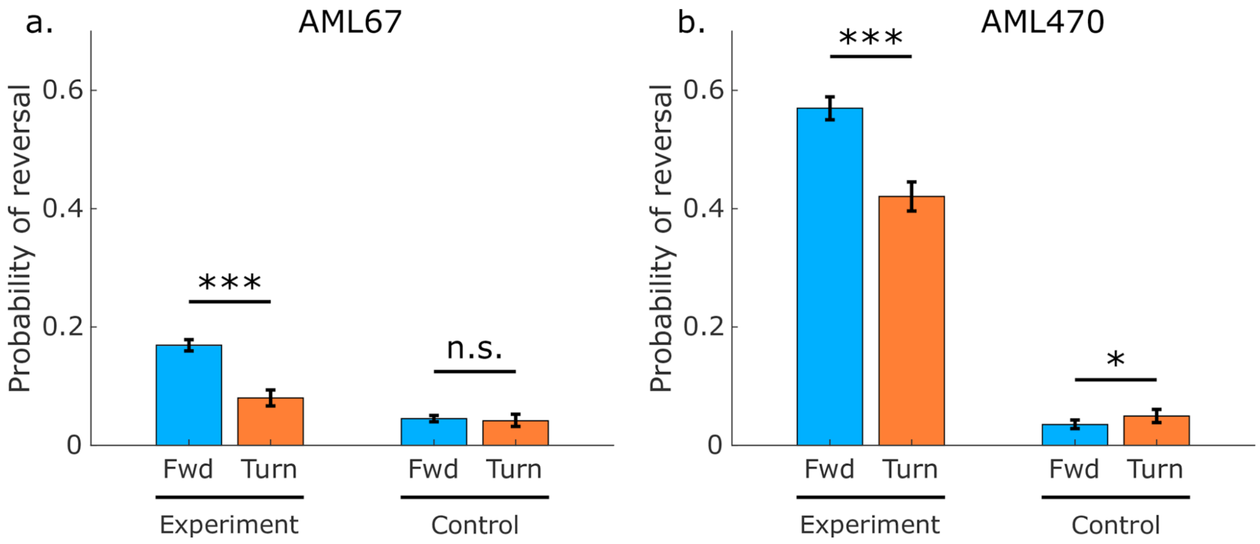

The choice of AML67 for this work was in part historical. We had already made crosses using AML67, such as the one in Fig 4 (AML499), before we generated AML470. Therefore, for consistency, we stuck with AML67.

Supp Fig. 1: mec-4p:chrimson, promotor italics
Fixed.

Supp. Fig. 2: N =1,631 and 10,94 stim events; a number seems missing.
Fixed.

Supp Fig 3: In contrast to all other graphs, the min/max values are 0/1. Why? I suggest to be consistent, unless there is a strog reason.
These are control experiments that correspond to the experiments in Fig. 2B. Therefore we set the y-axis limits to match those in Fig 2B to facilitate visual comparison. We now clarify in the caption:

"These are control experiments corresponding to the experiments presented in Fig. 2"

Reviewer #3:

Reviewer #3: Kumar et al investigate how animals integrate their responses to sensory stimuli with their current behavior. As a paradigm they study processing of information from mechanosensory neurons in C. elegans. C. elegans navigate their environment by alternating between persistent forward and reversals and turns. Aversive stimuli, such as anterior touch, evoke reversals during which the animal backs away from the stimulus, then turns sharply. This behavioral sequence usually leads animals to locomote away from the aversive stimulus. However, for this escape behavior to be successful, animals need to suppress recurrent reversals after the turn to allow them to move away from the noxious cue.

The authors begin by showing that stimulating mechanosensory neurons during a turn is significantly less likely to evoke a reversal than equivalent stimulation when animals are moving forward. They perform this experiments using sophisticated closed loop optogenetic stimulation of gentle touch mechanosensors. Previous work identified a set of neurons that promote reversal behavior that include AIZ, RIM, AIB, AVE and AVA. Most of these neurons are one or two synapses downstream of the anterior touch neurons. The authors use the same strategy of closed loop optogenetics to stimulate each of these neurons in turn while animals are moving forward or turning. Again, they find that stimulating these neurons in turning animals is less likely to evoke a reversal than stimulating them while animals are moving forward. An exception is the AVA neurons, leading the authors to infer that AIZ, RIM, AVD and AVE neurons are at or upstream of the junction where turning circuits inhibit
reversal behavior, whereas AVA is downstream They next show that inhibiting neurons previously implicated in turning behavior, namely RIV, SMB and SAA, prolongs the duration of spontaneous reversals. Finally, they show that optogenetically stimulating mechanosensory neurons when animals are moving forward or turning evokes similar rates of reversals if RIV, SAA and SMB neurons are inhibited during turns.This then suggests a model in which neurons that are active during turning (RIV, SMB and SAA) inhibit neurons that promote reversal (AIZ, RIM, AIB, AVE).

The optogenetic approach used by the authors is powerful, and the data they provide supports their circuitry model in which neurons that promote turning inhibit neurons that promote reversal behavior. The usual caveats apply that optogenetic stimulation may not accurately reproduce physiological circuit activity, but the authors have sought to control this as much as possible. The logical next step is to elucidate the molecular mechanisms mediating inhibition of reversal neurons by turning ones. The authors outline one such potential mechanism, mediated by inhibitory acetylcholine receptors,

in the discussion, and cite complementary work recently submitted to Biorxiv by Huo and colleagues (citation 48) that provide data supporting this mechanism. In summary, the experiments described are well-designed, and the manuscript is clearly written, concise, and easy to understand. I support publication.

We thank the reviewer for their thoughtful feedback.

Reviewer #4

Reviewer #4: "Inhibitory feedback from the motor circuit gates mechanosensory processing in C. elegans" by Kumar et al. uses their previously published method to activate or inhibit particular neurons in worms triggered by particular behaviors. Their previous paper had shown, among other things, that worms' sensitivity to optogenetic activation of the touch neurons depended on their behavioral state. If they were in the process of turning, their probability of reversing in response to the simulated touch was reduced. In the current paper they reproduce this finding with more data and show that isolated turns (initiated during forward locomotion) and escape turns (turns following a reversal) both 'gate' the response to touch neuron stimulation.

The main new findings result from experiments stimulating and inhibiting specific interneurons during the same behavior to identify which are involved in the gating. The authors find that stimulating specific interneurons (AIZ, RIM, AIB, AVE, and AVA) can cause reversals and in all cases except AVA, the probability of an evoked reversal was lower when the worms were turning. They further find that inhibition of turning-associated neurons (RIV, SMB, and SAA simultaneously) increased the length of spontaneous reversals. Finally, they show that inhibiting these neurons rescues the decrease in reversal probability upon optogenetic activation of the touch neurons during reversals. I.e. worms with inhibited RIV, SMB, and SAA reverse at the same rate whether they are turning or going forwards when their touch neurons are activated.

Together these results provide insight into the neural circuits involved in their previous observation of behaviour-modulated sensory responses. The paper is straightforward, well-written, and clear and makes a useful further contribution on top of the original paper.

I have only two minor comments:

-Was expression in the appropriate neurons confirmed for all strains? Especially for the new strains, it would be good to include some supplementary images showing the expression pattern.

We have now added new Supplementary Fig S5, pasted below, that shows expression patterns for the new RIV, SMB and SAA strain, described in response to Reviewer #1

comment #3 above. Expression matches expectation and previous reports in Wang et al., eLife 2020.

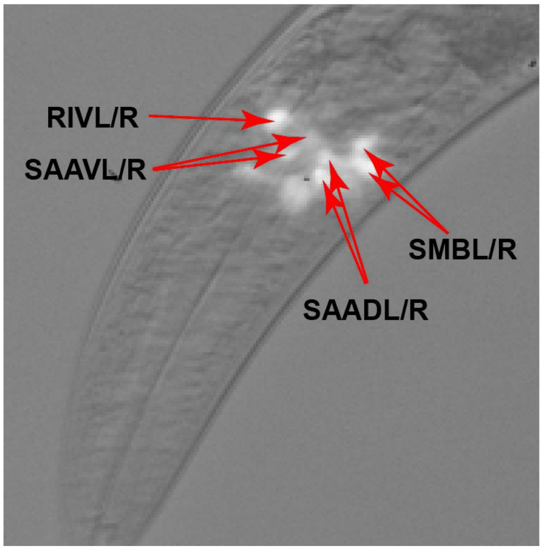

We also added the corresponding text:

> *"To express gtACR2 in RIV, SMB, and SAA, we used the* lim-4*, promoter, as in (Wang et al., 2020) and confirmed the expression pattern using fluorescence microscopy, Supplementary Figure S5."*

Other new strains are crosses of this strain. The rest of the strains in the work come from published sources.

-some gene names are not italicized such as lgc-47, acc-1, and acr-3 on page 9.

Thank you. Fixed.
